# Vegetarian Diets and Medical Expenditure in Taiwan—A Matched Cohort Study

**DOI:** 10.3390/nu11112688

**Published:** 2019-11-06

**Authors:** Chin-Lon Lin, Jen-Hung Wang, Chia-Chen Chang, Tina H.T. Chiu, Ming-Nan Lin

**Affiliations:** 1 Department of Cardiology, Dalin Tzu Chi Hospital, Buddhist Tzu Chi Medical Foundation, Chiayi County 622, Taiwan; 2 Department of Internal Medicine, College of Medicine, Tzu Chi University, Hualien 970, Taiwan; 3 Department of Medical Research, Buddhist Tzu Chi General Hospital, Hualien 970, Taiwan; jenhungwang2011@gmail.com (J.-H.W.);; 4 Department of Nutritional Science, Fu-Jen Catholic University, Taipei 242, Taiwan; tina925@gmail.com; 5 Department of Family Medicine, Dalin Tzu Chi Hospital, Buddhist Tzu Chi Medical Foundation, Chiayi County 622, Taiwan; 6 Department of Family Medicine, College of Medicine, Tzu Chi University, Hualien 970, Taiwan

**Keywords:** medical expenditure, vegetarian diet, lifestyle, Buddhist

## Abstract

Vegetarian diets and lifestyle have been shown to reduce the risk of many chronic non-communicable diseases, which now accounts for the major global burden of diseases. We aimed to determine the contribution of vegetarian diets and lifestyle to the actual direct medical cost in a population-based study. Through linkage to the National Health Insurance Research Database of Taiwan, we compared the health care utilization and medical expenditure of 2166 vegetarians and 4332 age-sex-matched omnivores recruited from the Buddhist Tzu Chi Foundation. Diet and lifestyle questionnaires were self-administered and prospectively collected. We used the general linear model to estimate the 5-year average medical expenditure in vegetarians versus omnivores while adjusting for age, sex, education, exercise habits, smoking, and alcohol drinking. Medical expenses related to non-diet associated lifestyle factors (smoking, alcohol drinking, active community volunteering, and religious emotional support) were estimated through a comparison with the published population medical cost data standardized to match the age and sex characteristics of the cohort. Tzu Chi vegetarians had significantly lower outpatient visits. This translated into 13% lower outpatient (*p* = 0.007) and 15% lower total medical expenditures (*p* = 0.008) when compared with the Tzu Chi omnivores, who had an additional 10% lower medical expenditure when compared with the general population. No difference in dental visits and expenses were found between diet groups. Vegetarian diets are associated with significantly lower medical care expenditure and could be an effective strategy to alleviate the medical–economic burden in selected populations.

## 1. Introduction

In the Global Burden of Disease Study, population growth, ageing, and decreased death rates shift the disease patterns from communicable, maternal, neonatal, and nutritional causes toward non-communicable diseases [1]. The biggest contributors to the world’s health burden are now mostly chronic diseases and injuries. In Taiwan, the top causes of death and disability are diabetes, chronic obstructive pulmonary diseases, stroke, and ischemic heart diseases in 2017 [2]. The leading risk factors for global disease burden are now mainly lifestyle-related: tobacco smoking, alcohol use, high blood pressure, high fasting blood glucose, high body mass index [1,2,3]. Cigarette smoking [4,5,6], excessive alcohol drinking [7,8], physical inactivity [9,10,11], unhealthy diets [12,13,14], overweight/obesity [15,16], and other lifestyle factors [7,17] have all been independently shown to increase the risk of certain diseases and their associated healthcare utilization and expenditure.

Vegetarians typically consume more grains, legumes, vegetables, fruits, and nuts [18]. Vegan diets include only plant foods, while lacto-ovo-vegetarian diets include dairy and/or egg products. The health benefits of plant-based diets have been studied extensively. Individuals following a plant-based or a vegetarian dietary pattern typically have lower cholesterol levels [19], lower blood pressures [20], and lower BMI [21]. These place them at a lower risk for many diseases including heart disease, diabetes, cancer, hypertension, gout, gallstone disease, and nonalcoholic fatty liver [22,23,24,25,26,27,28].

Vegetarian diets, by exerting protection against certain diseases, may be an effective strategy to reduce medical care utilization and expenditure, but the actual amount is difficult to calculate since other factors associated with a vegetarian lifestyle (i.e. smoking, alcohol consumption, physical activity, and body weight) might also play significant roles. Several studies have attempted to estimate the medical cost related to meat consumption or vegetarian diets by published relative risk of common chronic diseases [12,17], but the actual direct contribution of vegetarian diets to the reduction in healthcare utilization and expenditure remain to be determined.

This study aims to examine the actual direct contribution of vegetarian diets and lifestyle to healthcare utilization and expenditure in Taiwan. Effects of a vegetarian diet vs. an omnivorous diet on healthcare costs were analyzed by linking a non-smoking, non-alcohol drinking community-based Buddhist cohort (one-third are vegetarians) to the National Health Insurance Research Database. In addition, we compared the cohort participants’ medical expenditure to those of the general population to estimate expenditure related to other lifestyle factors. We hypothesized that vegetarians incur a lower medical expenditure due to lower risks for chronic non-communicable diseases. 

## 2. Materials and Methods 

### 2.1. The Tzu Chi Vegetarian Study

The Tzu Chi Vegetarian Study (TCVS) is a prospective cohort established to investigate the association between vegetarian diets and chronic disease incidences. Thus far, the cohort has published its results on the incidence of gout [28]. The TCVS recruited 12,062 Buddhist volunteers from the Buddhist Tzu Chi Foundation in 2005. The Foundation is well known for its high number of devoted volunteers who dedicate themselves to charity work, community services, recycling and environmental conservation, and in disaster relief in both Taiwan and international settings. All volunteers are organized at community levels and meet frequently for various projects. The Foundation requires all its certified volunteers to observe the 10 precepts of Buddhism, which prohibit smoking and alcohol drinking. In addition, volunteers are encouraged to consume a vegetarian diet or to cut down meat consumption, as a way to combat climate change and to be kind to animals. About one-third of the cohort follow a vegetarian diet (mostly ovo-lacto-vegetarians, few are vegans who also avoid eggs and milk products). Most volunteers quit smoking and alcohol drinking before becoming certified as Tzu Chi volunteers.

The Buddhists Tzu Chi Foundation has many sites throughout Taiwan. In 2005, community leaders in all Tzu Chi sites were invited to distribute research questionnaires to their local community volunteers, and collect and send these questionnaires back upon completion. The study was approved by the Institutional Reviewer Board at the Dalin Tzu Chi Hospital (IRB number: B10403020). Signed informed consent was obtained from all study participants (first page of the questionnaire).

### 2.2. Assessment of Healthcare Utilization and Expenditure

In Taiwan, the National Health Insurance (NHI) Program is a government sponsored universal health insurance program that covered 96% of the population in 2001 and expanded to 100% coverage by 2010 [29,30]. The NHI benefits included inpatient and outpatient care, pharmaceuticals, dental care, and catastrophic illness, and requires copayments (typically 10–15%) by users (waived for those defined as poor). The actual medical insurance claim data are available through the Health and Welfare Data Science Center (HWDSC), Ministry of Health of Taiwan, for research purposes. We linked the cohort participants’ baseline data to the medical claim data and mortality data at the HWDSC through a unique personal identification number. We retrieved the medical cost of participants from July 2005 to July 2010 and calculated the annual average medical expense for each participant. In order to protect the privacy of individuals, all analyses were performed within the HWDSC and only summarized research finding (no individual data) could be released. Since almost all medical services and pharmaceuticals (except cosmetic surgery, dentures, over the counter vitamins, etc.) are covered by the NHI, our data reflect the total direct healthcare expenses incurred by the population.

### 2.3. Assessment of Diet and Other Covariates

At enrollment, the participants filled out a questionnaire with sections on demographic information, medical histories, and lifestyle habits including smoking, alcohol drinking, physical activities and diet. The diet section was adopted from the Food Frequency Questionnaire (FFQ) used in the Nutrition and Health Survey in Taiwan (NAHSIT), and included 57 items on food or food groups, and 10 items on cooking methods and cooking oil. A similar questionnaire was later validated among Tzu Chi volunteers and showed good reliability and validity [31]. Vegetarians were defined as individuals who reported consuming meat, fish, and seafood less than once a month, while those consuming these foods greater than once per month were classified as omnivores in our study. Exercise habit was inquired in one question within the lifestyle section of the questionnaire, where exercise was defined as at least twenty minutes of exercise with sweating. Four choices included (1) every day, (2) at least three times per week, (3) occasionally, or (4) no exercise habit. Those reporting (1) to (3) were classified as “yes” and those reporting (4) were classified as “no” for exercise habit.

### 2.4. Statistical Analysis

**Figure 1:**
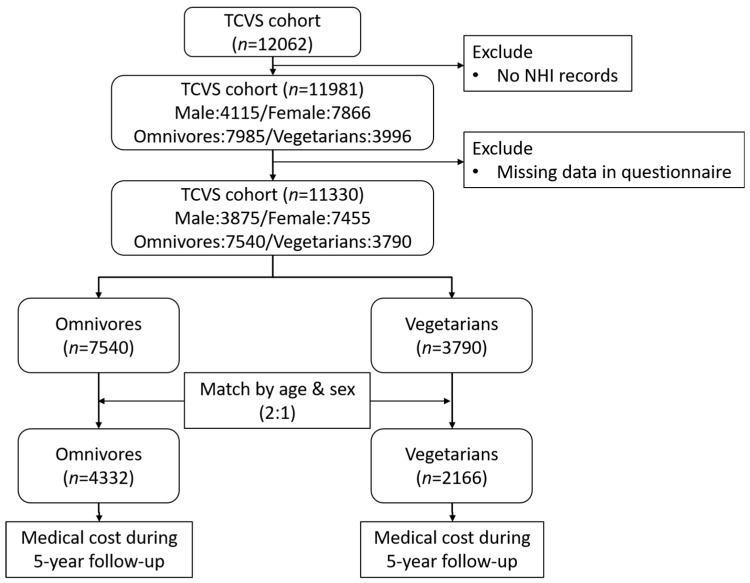
The study protocol.

The study protocol is shown in Figure 1. Of the 12,062 volunteers who returned their questionnaire, 11,981 (male 4115, female 7866) provided valid personal identification numbers to link to the NHI database. Among them, 651 were excluded due to missing data (such as date of birth, sex) in the questionnaire. Of the remaining 11,330 (3875 male, 7455 female; 7540 omnivores, 3790 vegetarians) participants, we found a large discrepancy in age and sex between vegetarians and omnivores (vegetarians tended to be older with a higher proportion of females). Therefore, each vegetarian was matched with two omnivores by age and sex. Vegetarians without matched omnivores (no one with the same age and sex) were excluded from the analysis. All participants included were followed from the enrollment date until the earliest occurrence of death, dropout from the insurance program, or to the date of the five-year follow-up. The least square means of annual medical expenditure were computed using the general linear model with adjustment for age, sex, smoking, alcohol drinking habits, education, and exercise habits. All statistical analyses and graphics were performed in SAS V9.4 (SAS Institute, Cary, NC, USA). A 2-sided *p* < 0.05 was considered statistically significant. To estimate the medical expenditure from the Taiwanese population, we used the medical cost statistics from the official website of the Taiwan Ministry of Health and Welfare [32]. Population average medical costs were calculated by standardizing costs to match the age and sex composition of the selected Tzu Chi population, in order to generate a comparable estimate of medical expenditure for the general population. We used Stata statistical software (Release 14, STATA Corp, College Station, TX, USA) for power calculations. The Stata command power two means can be used to estimate power for two-sample data for a sample of at least 5523 (1841 in vegetarian group and 3682 in omnivore group), an effect size of 0.08, and an α of 0.05 with a 2-sided test were set in STATA software. The statistical power was estimated to be more than 80% and would be able to detect any significant difference in the two groups.

## 3. Results

Table 1 shows the baseline characteristics between vegetarians and matched omnivores. Age and sex were the same due to the matching process. Vegetarians were slightly less likely to have an exercise habit. Education levels and marital status were similar between vegetarians and omnivores. Very few people reported the use of cigarettes or alcohol in both vegetarians and omnivores.

**Figure 2:**
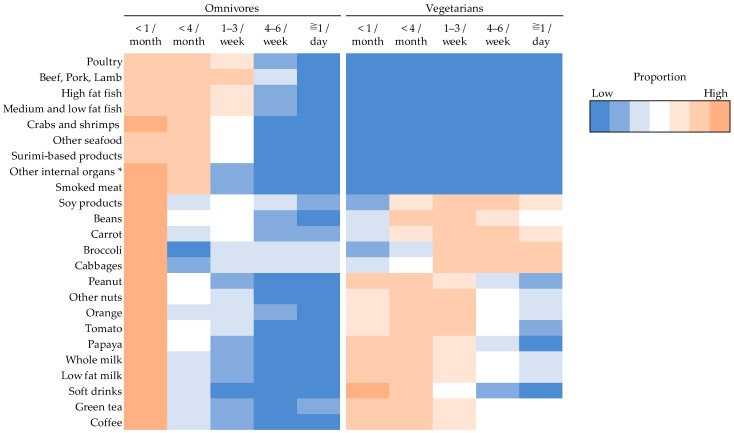
Heatmap of food intake in omnivores and vegetarians. Rows: food items; Columns: frequency of food intake in two groups. * Other internal organs include kidney, intestines, stomach, heart.

Dietary intake (selected items in the FFQ) of the omnivores and vegetarians are shown in Figure 2. The heatmap visualization shows distinct patterns of food intakes in omnivores and vegetarians. The proportion of individuals in each frequency category of intake was indicated by the continuum of colors ranging from dark orange (high proportion) to dark blue (low proportion). Among the omnivores, consumption of each type of meat or fish concentrated in the frequency of 1–3 per week or less. Vegetarians reported eating no meat and fish, but had a high proportion of individuals consuming soy products and vegetables on a more frequent basis. Vegetarians also appeared to consume soy, fruits, vegetables, and nuts more frequently than omnivores. 

Table 2 shows the healthcare utilization and expenditure of vegetarians and omnivores. Vegetarians had a 15% lower total medical expenditure and a 13% lower outpatient medical expenditure when compared with omnivores. After further adjustment for age, sex, education, smoking, alcohol drinking, and exercise habits, the medical expense reduction associated with vegetarian diet strengthened slightly. Vegetarians also had a 20% lower medical expenditure associated with inpatient care, though this did not reach statistical significance. No difference was found for dental service expenditure between vegetarians and omnivores.

Table 3 shows the healthcare expenditure after stratifying by age and sex. The lower medical expenditure was consistent across the age group strata, though less significant in those aged ≥50. When examining subgroups by sex, we found that a larger discrepancy in medical cost between vegetarians and omnivores were found mainly in females. 

Table 4 lists the medical expenditures for the most common diagnosis. Vegetarian diet was associated with statistically significant lower medical expenditure in hypertension (28% reduction), dyslipidemia (31% reduction), and depression (48% reduction), and a non-significant lower expenditure related to coronary heart disease (22% reduction) and renal disease (84% reduction).

**Figure 3:**
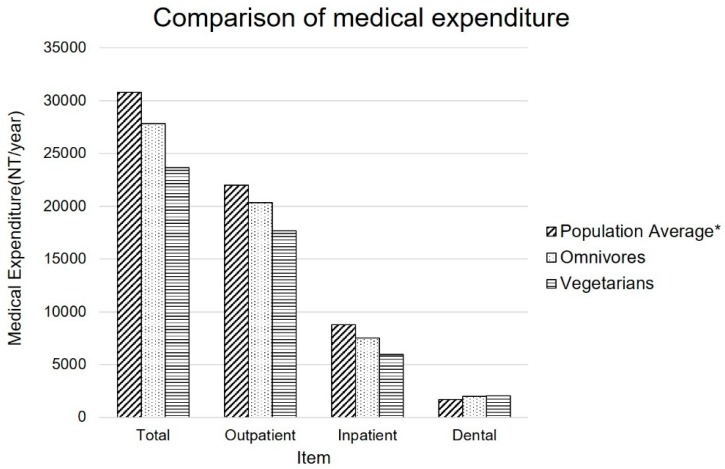
Medical expenditure of vegetarians and omnivores in the Tzu Chi Vegetarian Study, and the age-sex-standardized population average. OPD: Outpatient Department, IPD: Inpatient Department, Dental: Dental Department, * Estimated sex and age-standardized population average.

Figure 3 shows the graphic comparison of healthcare expenditure among the vegetarian and omnivore cohort participants and the age–sex-matched population average. Omnivore participants had 10% lower medical expenditure compared with the general population. Vegetarians had 15% lower total medical cost than omnivores and an approximately 25% cost reduction compared with general population.

## 4. Discussion

In this prospective study, we found that vegetarians had a significantly lower direct medical expenditure (15%) when compared with omnivores in our group. A reduction in the medical expenses was found for the treatment of several diet and lifestyle related non-communicable disease and conditions including hypertension, dyslipidemia, and depression. 

In our study, vegetarian dietary pattern (characterized by a more frequent consumption of fruits, vegetables, soy, and nuts, while avoiding meat) was associated with a lower total and outpatient medical expense than the omnivorous dietary pattern (characterized by a relatively more frequent consumption of meat and fish, and less frequent consumption of various plant-based foods). The contrast in the dietary patterns also translated into a difference in medical expenditure associated with hypertension, coronary heart disease, and depression. Large prospective US and European cohort studies and meta-analyses of epidemiological studies have indicated that the consumption of red meat is associated with an increased risk of total mortality, cardiovascular disease, colorectal cancer, and type 2 diabetes in both men and women [33]. The total direct medical costs attributable to meat consumption was estimated to be $28.6–64.1 billion in 1992, similar to the $50 billion estimated for cigarette smoking (7.1% of U.S. total direct medical expenditure) in 1993 [12]. Higher fruit and fruit plus vegetable intakes were associated with approximately 18% lower mean annual and cumulative Medicare charges in the United States [34]. Poor diet-related illness costs the NHS in the UK £5.8 billion, or 7.13% of total NHS budget in 2006–07 calculated using population attributable fractions (PAF) [33]. In Taiwan, the elderly who spent more on purchasing fruits and vegetables (highest tertile) had a reduced all-cause mortality, used less medical services, and incurred 19% lower total medical costs than those in the lowest tertile [35]. Our study results are consistent with other published studies and underscore the fact that dietary instructions (promotion of vegetarian diets) is a potential strategy to alleviate the current disease–economic burden in selected populations.

The omnivore group had an additional 10% reduction of medical expenses when compared with age and sex-matched estimates of the general population of Taiwan, probably as a result of the unique lifestyle of our cohort. Participants of this cohort are devoted Buddhists; they neither smoke cigarettes nor drink alcohol. These participants also volunteer actively in various community services such as recycling, helping the poor, and participating in disaster relief activities. The impacts of smoking and alcohol drinking on healthcare utilization and expenditure have been reported extensively. The annual medical cost related to smoking in the United States has been estimated to be 6–8% of the total personal healthcare expenditure [3]. A recent review estimated an even higher expenditure (8.7%) [6]. In Greece, a study using a prevalence-based annual cost approach found that 10.7% of the national hospital budget was attributable to smoking related illnesses in 2011 [5]. The estimated economic cost of excessive drinking was very high, mainly from lost productivity, criminal justice costs, etc., while direct healthcare costs accounted for around 11% of the total cost [8,36]. In the United Kingdom, 3–4% of the total National Health Service (NHS) expenditure could be attributable to excessive alcohol drinking [7,37]. Using the American Heart Association’s Life Simple 7 (LS7, metric includes seven factors: cigarette smoking, physical activity, diet, body mass index, blood pressure, cholesterol, and glucose) as the baseline metric for cardiovascular health, Aaron [17] demonstrated that better cardiovascular health metrics were associated with lower risk for inpatient encounters and lower inpatient and outpatient healthcare expenditures in participants over 65 years of age, by linking to Medicare claims data. Compared with those with higher risk factors, those with lower risk factors had potential annualized cost reductions of 53.4%, 29.7%, and 37.5%, for inpatient, outpatient, and total expenditures, respectively. The magnitude of the reduction in total healthcare expenditure (37.5%) for lifestyle was much higher than that in our study (10–23%). This can be explained, at least in part, by the age differences of the study population (>65 years of age as compared with our study group’s average age of 50 years). The total healthcare expenditure is much higher in those over 65 years of age than those under 65 [38]. Lifestyle has long lasting effects, and could potentially affect the risk of chronic diseases and influence the healthcare utilization and expenditure to a higher degree as population ages. We believe that the savings in medical expenditure will be even greater as the population ages. In addition to diet and lifestyle, participation in religion-related activity may potentially contribute to better health, and the lowering of depression and mortality [39]. The lower medical expenditure between participants of this religion-based cohort and the general population may be partly attributed to religious activities, though more research is needed to clarify this. 

Strength and Limitations: The strength of this study includes the prospective design to lessen recall bias, and the high follow-up rate due to the nearly complete coverage of the NHI which reduces selection bias. The availability of actual medical expense data enabled us to calculate the direct cost, rather than just estimations based on the relative and absolute risk of diseases. This study also demonstrated the unique contribution of a vegetarian diet to medical expenditure since the lifestyles between vegetarians and omnivores in our study were similar, except for dietary preferences. However, there were several limitations in our study: (1) lifestyle habits such as smoking, alcohol drinking, and physical activity were reported by the study participants and not measured objectively; (2) diet was assessed only at the baseline, and change over time is possible, though this would tend to attenuate the association toward the null; (3) actual medical utilization and expenditure could be influenced by healthcare seeking behaviors in which health conscious individuals may be more likely to seek medical care or detect diseases at an earlier stage, which may initially make them more “costly”, but should reduce long-term medical expenses; and (4) our study was based on a group of devoted Buddhists who abstain from alcohol and tobacco, and generalizability to the whole population requires confirmation from other studies.

## 5. Conclusions

In short, our study demonstrated that a healthy lifestyle (no smoking, no alcohol drinking) may reduce the total direct healthcare expenditure by 10% and vegetarian diets may further reduce medical care expenditure by 15% (therefore, approximately 25% reduction in total medical expenditure). These results are consistent with other published studies and suggest that the promotion of a vegetarian diet may be a hopeful strategy to reduce the medical–economic burden in selected populations.

## Figures and Tables

**Table 1:** Demographic and lifestyle characteristics of vegetarians and omnivores.

Item	Omnivores	Vegetarians
Number of subjects	4332	2166
Age	50.5 ± 9.4	50.5 ± 9.3
Age Group		
<50	2035 (47.0%)	1030 (47.6%)
≧50	2297 (53.0%)	1136 (52.4%)
Sex		
Male	1548 (35.7%)	774 (35.7%)
Female	2784 (64.3%)	1392 (64.3%)
Marriage		
No	372 (9.9%)	235 (10.8%)
Yes	3905 (90.1%)	1931 (89.2%)
Education		
Elementary school or less	1025 (23.6%)	481 (22.2%)
Secondary school	2277 (52.6%)	1124 (51.9%)
College or higher	1030 (23.8%)	561 (25.9%)
Smoking		
Never	3672 (84.8%)	1868 (86.2%)
Past	594 (13.7%)	290 (13.4%)
Current	66 (1.5%)	8 (0.4%)
Alcohol drinking		
Never	3713 (85.7%)	1861 (85.9%)
Past	523 (12.1%)	292 (13.5%)
Current	96 (2.2%)	13 (0.6%)
Exercise habits		
No	810 (18.7%)	464 (21.4%)
Yes	3522 (81.3%)	1702 (78.6%)

Data are presented as n (%) or mean ± standard deviation.

**Table 2:** Comparison of annual total medical, outpatient, hospitalization, and dental expenditure between omnivores and vegetarians. (N = 6498) in New Taiwan Dollar (NT).

Item	Crude				Adjusted ^†^			
	Omnivores	Vegetarians	Savings (%)	*p*-Value	Omnivores	Vegetarians	Savings (%)	*p*-Value
Total medical expenditure (NT/per year)	27,841 ± 976	23,656 ± 1381	15%	0.013 *	25,222 ± 3060	20,949 ± 3300	17%	0.011 *
Outpatient								
No. of visits (times/per year)	19.8 ± 0.23	17.6 ± 0.28	-	<0.001 *	-	-	-	-
Medical expenditure (NT/per year)	20,333 ± 607	17,679 ± 858	13%	0.012 *	17,802 ± 1900	15,073 ± 2049	15%	0.009 *
Inpatient								
No. of visits (times/per year)	0.14 ± 0.01	0.12 ± 0.01	-	0.149	-	-	-	-
Medical expenditure (NT/per year)	7508 ± 617	5978 ± 872	20%	0.152	7420 ± 1940	5876 ± 2092	21%	0.149
Dental								
No. of visits (times/per year)	1.80 ± 0.03	1.83 ± 0.04	-	0.566	-	-	-	-
Medical expenditure (NT/per year)	1995 ± 30	2059 ± 42	−3%	0.217	1749 ± 94	1814 ± 101	−4%	0.209

Data are presented as mean ± standard error. * *p*-value < 0.05 was considered statistically significant after test. ^†^ Adjusted for age, sex, education, smoking, alcohol drinking, and exercise habits.

**Table 3:** Comparison of annual total medical, outpatient, hospitalization, and dental expenditure between non-vegetarians and vegetarians after stratifying by age and sex in New Taiwan Dollars (NT).

Item	<50 years of age (N = 3065)			≧50 years of age (N = 3433)			Male (N = 2322)			Female (N = 4176)		
	Adjusted ^†^			Adjusted ^†^			Adjusted ^‡^			Adjusted ^‡^		
	Omnivores	Vegetarians	Saving (%)	Omnivores	Vegetarians	Saving (%)	Omnivores	Vegetarians	Saving (%)	Omnivores	Vegetarians	Saving (%)
Total medical expenditure (NT/per year)	19,325 ± 3375	15,445 ± 3662	20% *p* = 0.047 *	31,074 ± 5206	26,396 ± 5556	15% *p* = 0.081	25,880 ± 3951	23,151 ± 4599	11% *p* = 0.391	21,131 ± 6761	16,106 ± 6911	24% *p* = 0.010 *
Outpatient medical expenditure (NT/per year)	14,999 ± 2421	12,245 ± 2627	18% *p* = 0.049 *	20,315 ± 2995	17,608 ± 3196	13% *p* = 0.080	18,244 ± 2534	17,082 ± 2950	6% *p* = 0.569	17,234 ± 4081	13,736 ± 4171	20% *p* = 0.003*
Inpatient medical expenditure (NT/per year)	4325 ± 1784	3201 ± 1936	26% *p* = 0.275	10,759 ± 3508	8788 ± 3744	18% *p* = 0.276	7636 ± 2435	6069 ± 2834	21% *p* = 0.424	3897 ± 4384	2369 ± 4482	39% *p* = 0.226
Dental medical expenditure (NT/per year)	1700 ± 122	1783 ± 132	−5% *p* = 0.241	1791 ± 146	1841 ± 156	−3% *p* = 0.512	1797 ± 109	1939 ± 127	−8% *p* = 0.106	1622 ± 223	1648 ± 228	−2% *p* = 0.690

Data are presented as mean ± standard error. * *p*-value < 0.05 was considered statistically significant after test. ^†^ Adjusted for sex, education, smoking, alcohol drinking, and exercise habits. ^‡^ Adjusted for age, education, smoking, alcohol drinking, and exercise habits.

**Table 4:** Comparison of annual medical expenditure of common diseases between omnivores and vegetarians.

Disease	Omnivores	Vegetarians	Savings (%)	*p*-Value
Hypertension	3819 ± 683	2762 ± 737	28%	0.005 *
Diabetes	2529 ± 643	2079 ± 694	18%	0.209
Dyslipidemia	1333 ± 273	914 ± 295	31%	0.006 *
Cerebrovascular disease	561 ± 943	252 ± 1018	55%	0.557
Renal disease	1831 ± 1568	305 ± 1692	84%	0.081
Coronary heart disease	1995 ± 430	1553 ± 464	22%	0.065
Depression	794 ± 254	410 ± 274	48%	0.007 *

Data are presented as mean ± standard error. Unit: NT/per year * *p*-value < 0.05 is considered statistically significant after the test. Model: Adjusted for age, sex, education, smoking, alcohol drinking, and exercise habits.
